# Resonances of nanoparticles with poor plasmonic metal tips

**DOI:** 10.1038/srep17431

**Published:** 2015-11-30

**Authors:** Emilie Ringe, Christopher J. DeSantis, Sean M. Collins, Martial Duchamp, Rafal E. Dunin-Borkowski, Sara E. Skrabalak, Paul A. Midgley

**Affiliations:** 1 Department of Materials Science and NanoEngineering, Rice University, 6100 Main St., Houston TX 77005, USA; 2 Department of Chemistry, Indiana University, 800 E. Kirkwood Ave., Bloomington, IN 47405, USA; 3 Department of Materials Science and Metallurgy, University of Cambridge, 27 Charles Babbage Road, Cambridge CB3 0FS, UK; 4 Ernst Ruska-Centre for Microscopy and Spectroscopy with Electrons (ER-C) and Peter Grünberg Institut 5 (PGI-5), Forschungszentrum Jülich GmbH, D-52425 Jülich, Germany

## Abstract

The catalytic and optical properties of metal nanoparticles can be combined to create platforms for light-driven chemical energy storage and enhanced *in-situ* reaction monitoring. However, the heavily damped plasmon resonances of many catalytically active metals (e.g. Pt, Pd) prevent this dual functionality in pure nanostructures. The addition of catalytic metals at the surface of efficient plasmonic particles thus presents a unique opportunity if the resonances can be conserved after coating. Here, nanometer resolution electron-based techniques (electron energy loss, cathodoluminescence, and energy dispersive X-ray spectroscopy) are used to show that Au particles incorporating a catalytically active but heavily damped metal, Pd, sustain multiple size-dependent localized surface plasmon resonances (LSPRs) that are narrow and strongly localized at the Pd-rich tips. The resonances also couple with a dielectric substrate and other nanoparticles, establishing that the full range of plasmonic behavior is observed in these multifunctional nanostructures despite the presence of Pd.

Nanoparticles of free-electron metals such as Au, Ag, Cu, and Al can sustain narrow and intense localized surface plasmon resonances (LSPRs), light-driven coherent oscillations of conduction electrons. LSPRs and related phenomena are utilized in fields ranging from biological sensing to cancer therapy^1^,^2^,^3^,^4^. By incorporating dielectric shells, layering different metals, or creating heterogeneous aggregates, applications have been extended to, for example, surface-enhanced Raman scattering (SERS) tags and hydrogen sensors^5^,^6^. Stellated and multi-metal nanoparticles are of tremendous interest because of the superior electric field enhancement properties of their sharp tips, which intensify the resonance and refractive index sensitivity of the LSPRs^7^,^8^, and their potential for multifunctional behavior and novel properties. Unlike in the well-studied AgAu plasmonic alloys and core-shell structures^9^,^10^, the incorporation of the poor plasmonic metals Pd and Pt^11^,^12^,^13^ in multi-metal systems, while catalytically desirable, could be detrimental to the quality of the LSPR. Pd nanostructures indeed display broad, damped resonances when compared to their Au or Ag counterparts because of the presence of interband transitions through the visible spectrum^14^,^15^,^16^. Far-field optical studies have shown LSPR signatures as well as SERS activity in Pd-containing alloy nanoparticles^8^,^17^,^18^, but no direct observation of the fields around Pd have been reported.

Here we show that Au alloys containing a poor plasmonic metal^13^,^14^ (Pd) can nevertheless sustain multiple, size-dependent plasmon resonances and generate strong local field enhancement at the Pd-rich tips, where the composition is in fact the least favorable for plasmons. These Au/Pd stellated nanocrystals are also involved in substrate and interparticle coupling, as unraveled by nanometer spatial resolution electron energy loss spectroscopy (EELS), energy dispersive X-ray spectroscopy (EDS), and cathodoluminescence (CL) mapping. By establishing that the full range of plasmonic characteristics expected of Au is maintained in Au-Pd alloys, this study establishes the feasibility and provides strong motivation for research in multifunctional plasmonic-catalytic systems.

## Results

### Octopod shape, crystallography, and composition

**Figure 1:**
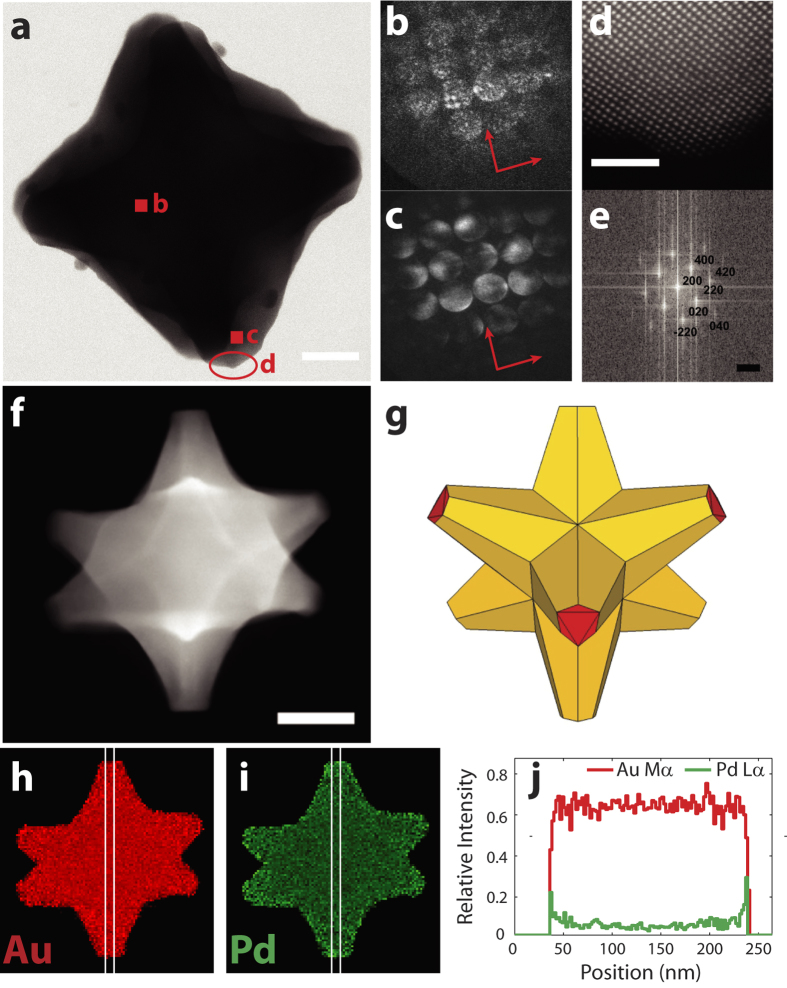
Structure, crystallography, and composition of Au/Pd octopods. (**a**) STEM bright field image. (**b,c**) Convergent beam electron diffraction (CBED) patterns obtained at the positions indicated in **a**, <001> orientation; the arrows indicate the orientation correspondence between **b** and **c**. (**d,e**) HAADF-STEM image of the region shown in a and associated Fourier transform, <001> orientation. (**f**) HAADF-STEM image of an octopod tilted −30°. (**g**) Model of an octopod tilted −30°. (**h**) Au M_α_, L_α_, and L_β_ summed relative X-ray intensity map. (**i**) Pd L_α_, L_β_, and K_α_ summed relative X-ray intensity map. (**j**) Relative X-ray intensity linescan of the Au M_α_ and Pd L_α_ lines along the vertical axis in (**h,i**). Scale bars, 25 nm for **a**, 2 nm for **d**, 5 nm^−1^ for **e**, 50 nm for **f,h,i**.

During Au/Pd co-reduction synthesis, overgrowth of seed Au crystals in the <111> directions creates 8-branched alloyed structures with point group symmetry O_h_ called octopods^19^. The well-defined protrusions are terminated by flat {111} facets, seen as sharp edges in the scanning transmission electron microscopy (STEM) images (shown in red in Fig. 1g) and electron tomograms (examples in Supplementary Figs 1–2 and Supplementary Movie 1). This stellated external morphology is, rather surprisingly, formed by a twin-free structure, as evidenced by nanometer resolution diffraction mapping (Fig. 1 and Supplementary Movie 2). The convergent beam electron diffraction (CBED) patterns in Fig. 1b,c show diffraction disks from two different areas of the particle, while Supplementary Movie 2 tracks the CBED patterns across the entire nanoparticle. All the CBED patterns acquired (Fig. 1b,c are two examples) are oriented along the same axes and have the same four-fold symmetry attributable to the <100> orientation in an FCC material at any position of the sub-nanometer probe. The consistency of orientation and symmetry in the diffraction map implies consistency in lattice orientation, i.e. each region of the octopod has a similar crystallographic orientation, an observation consistent with the lack of twinning in the structure. The single crystalline nature of the seeds is conserved through the synthesis, and the fully miscible Au and Pd form a continuous solid solution through the tips of the particle, rather than a patchy or polycrystalline core-shell structure, as shown by atomic resolution imaging (Fig. 1 and Supplementary Fig. 3). Diffraction patterns, Fourier transforms of lattice images, and atomic spacing measurements all yield a lattice spacing between that of Au and Pd, i.e. between 408 and 398 pm, also consistent with a solid solution, within the measurement error of a few percent. This lattice continuity, surface smoothness, and lack of scattering defects in Au/Pd particles are likely to have a favorable impact on the quality and lifetime of the plasmon resonances^20^.

STEM-EDS maps and linescans confirming Pd-rich tips^21^ were obtained by tilting the sample 30° so that most of the branches (6 out of 8) were isolated and directly addressable (Fig. 1 and Supplementary Figs 4–6). The co-reduction synthesis is likely the origin of this very steep gradient of Pd as Au deposits at a faster rate than Pd on the Au seeds and is thus depleted during growth^21^, a process also observed with Pt alloys^22^. Post-synthesis segregation is not expected thermodynamically since the surface free energy of Pd is higher than that of Au, at least in a clean environment^23^.

### LSPR mapping with EELS and CL

Light-matter interactions such as LSPRs can be probed in both the near-field and far-field. The bulk extinction spectrum, in ethanol, of our octopod sample has a single LSPR band centered at 800 nm and a high extinction coefficient of 9.8x10^11^ M^−1^ cm^−1^. However, this mode of analysis yields results on scattering, absorption, or extinction that are intrinsically diffraction-limited. The spatial resolution needed to understand plasmon mode distribution and symmetry can be achieved with electron microscopy-based techniques, which can simultaneously investigate size, composition, shape, and LSPRs in nanoparticles^24^,^25^,^26^,^27^,^28^,^29^,^30^,^31^,^32^,^33^,^34^.

Indeed, in addition to well-known imaging and elemental analysis techniques, the magnitude and energy of the interaction of the electron beam with plasmons can be probed by tracking the energy lost by electrons via EELS or by collecting the light emitted in the far-field as a result of plasmon decay via CL spectroscopy^26^,^28^,^33^. Simple shapes such as triangles, rods, shells, icosahedra and nanocubes^5^,^24^,^25^,^27^,^34^,^35^,^36^ have been studied, though to the best of our knowledge the near-field of alloy nanocrystals containing a poor plasmonic metal has never been mapped with sufficient resolution to unravel multiple modes and substrate effects.

**Figure 2:**
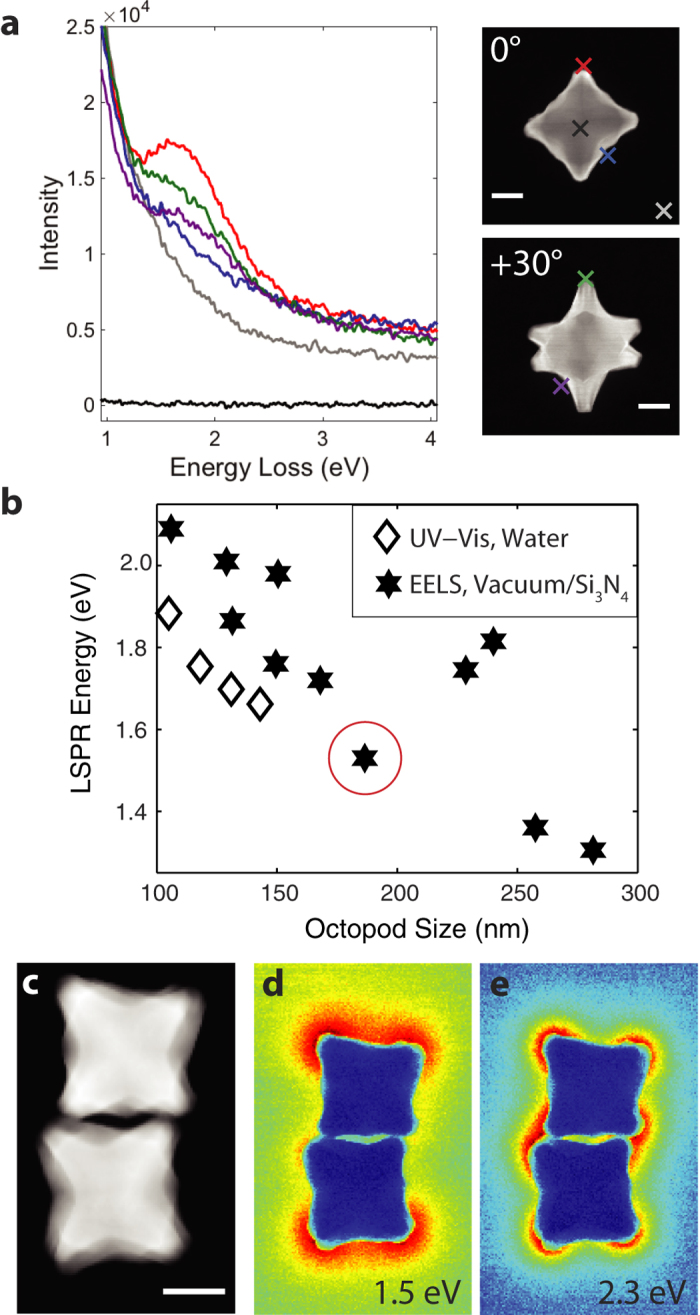
EELS of Au/Pd octopods, size dependence of the LSPR energy and plasmon coupling. (**a**) Monochromated STEM-EEL spectra at the different positions marked on the right HAADF-STEM images for the same particle tilted 0° and +30°. (**b**) Correlation between size (face diagonal) and energy of the lowest energy LSPR; the particle shown in **a** is circled in red. UV-Vis data from ref. 8 (**c**) HAADF-STEM image of an octopod dimer. (**d,e**) Reconstructed EFTEM images with a 0.2 eV slit centered at 1.5 and 2.3 eV. Scale bars, 50 nm.

The plasmonic behavior of 11 isolated octopods and several aggregates was analyzed using STEM-EELS tilt series; spectra from a particle at 0° and +30° are presented in Fig. 2. When the sub-nanometer beam is far from the particle, the signal obtained is simply the energy spread of the incoming electrons (the zero-loss peak, ZLP), while spectra obtained with the beam close to the particle tips display a broad but intense peak around 1.5 to 3.0 eV. Reconstructed energy filtered transmission electron microscopy (EFTEM) images obtained around the main spectral feature in single and aggregated nanocrystals indicate that a high EEL probability is concentrated at the sharp tips and that nearby particles interact (Fig. 2 and Supplementary Fig. 7). The dominant, bright bonding mode in the dimer occurs at 1.5 eV and the dark antibonding mode, at 2.3 eV, respectively lower and higher in energy than the expected LSPR energy for single octopods of this size (~1.8–2.0 eV), consistent with plasmon hybridization^37^.

**Figure 3:**
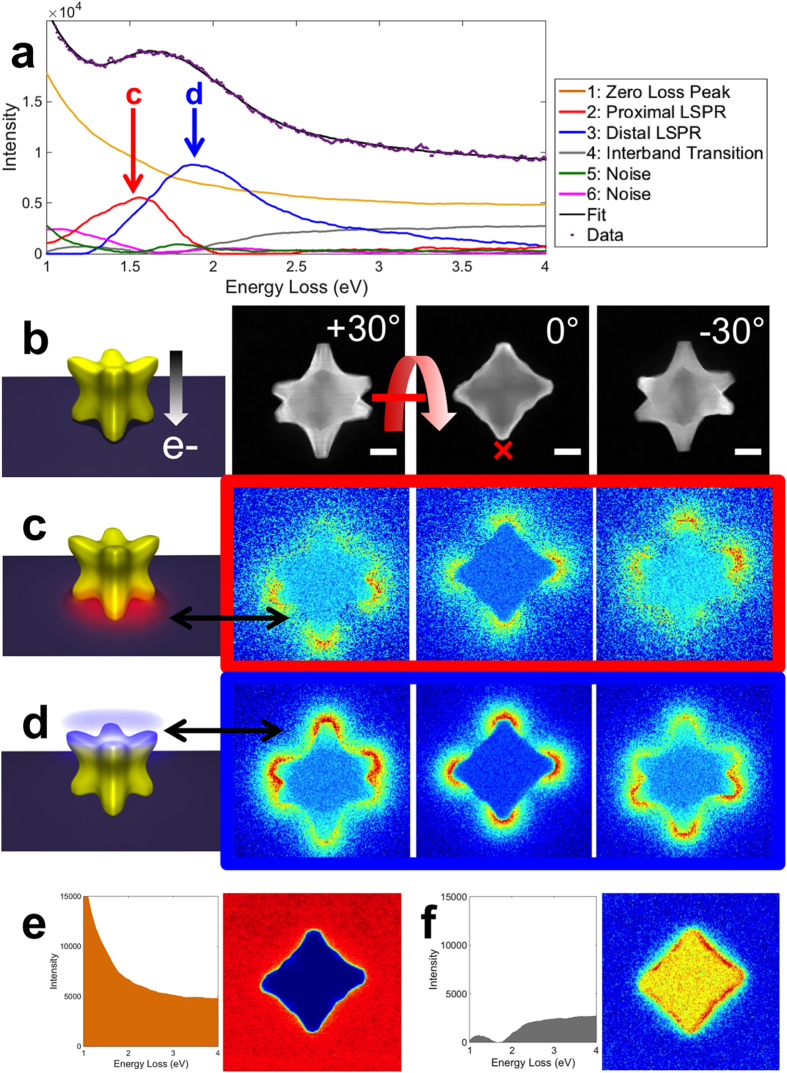
Statistical analysis of EELS results on a single Au/Pd octopod. (**a**) NMF spectral factors (plasmon modes and other contributions) and fit of the raw data using the EELS response at the position marked by a “x” in b. (**b**) Structural model of the 8-branched nanocrystal and dark field STEM images at +30, 0, and −30°. (**c**) Schematic and loadings for the proximal LSPR, representing the contribution of the mode to the overall EELS probability. (**d**) Schematic and loadings for the distal LSPR. (**e**) Spectral factor and loading at 0° for the tail of the zero loss peak. (**f**) Spectral factor and loading at 0° for the interband transition. (Full loadings are available Supplementary Fig. 8.) Scale bars, 50 nm. The EELS and STEM images have the same scale for each tilt.

Contributions from the tail of the ZLP and the unavoidable spectral and spatial overlap of high order modes make direct analysis of the raw spectra or reconstructed EFTEM images of single particles of limited use. To overcome this difficulty we used a blind source separation technique (non-negative matrix factorization, NMF) to extract individual plasmon resonances in single octopods and decouple the effects of the excitation energy spread (Figs 2 and 3 and Supplementary Figs 8–9)^24^,^38^. This approach has recently proven useful to extract plasmonic modes in nanorods, bipyramids, and nanocubes^24^,^39^,^40^.

The size tunability of the plasmon resonance is readily addressable from electron microscopy as high angle annular dark field (HAADF) images can be acquired concurrently with the EELS spectrum image (SI), providing size and LSPR energy for each particle studied. This relationship is shown in Fig. 2 for the lowest energy mode, a proximal resonance coupled with the substrate (*vide infra*). The peak position reported is the maximum of the decomposed spectral component rather than the maximum of the entire EELS spectrum. This distinction is critical: the latter is inevitably shifted due to the tail of the ZLP and overlapping resonances, while the maximum of a decomposed resonance only contains contributions from a specific LSPR.

The maximum of the extinction spectrum of bulk Au/Pd octopods was previously observed to vary nearly linearly from 2.19 to 1.66 eV when the average distance between opposing branches (size) increased from 61 to 143 nm, as expected from retardation effects. EELS analysis shows a decrease from 2.1 to 1.5 eV when the particle size increases from 106 nm to 282 nm, a similar trend with an offset fully justifiable on the basis of the different refractive index environments (see Supplementary Discussion). The two apparent outliers in Fig. 2 are attributed to the heavy oxygen/argon plasma cleaning these two particles were uniquely subject to. Removing carbon by plasma cleaning (as can be observed by EELS) leads to a downward shift of the refractive index resulting in a blue shift of the LSPR energy^41^. The correspondence between EEL and extinction spectroscopy shows that the very tips of the particles act as refractive index sensors, a conclusion not directly achievable in bulk extinction measurements. Moreover, this unique and novel approach to correlate a mode-specific energy with particle size for many particles, enabled by using a consistent number of components for all the particles analyzed with NMF, greatly facilitates the study of structure-function relationships such as the size effects presented in Fig. 2.

The near-field plasmonic behavior of Au/Pd nanocrystals was unraveled at the nanometer scale using statistical analysis (NMF) of a STEM-EELS tilt series (Fig. 3). Spectral factors including the tail of the ZLP, plasmon modes, and other energy loss contributions were extracted. Those factors are multiplied at each pixel by unique, position dependent loadings to generate a global fit, i.e. a linear combination of the factors reconstructs each spectrum in the SI. Mapping of the loadings hence provides the spatial distribution, i.e. contribution to the overall EEL probability, of a given spectral factor. The spectral fit matches the raw data well; the 6 spectral factors extracted fully explain the observed variations in the SI. In Fig. 3a, two narrow spectral factors (2 and 3) can be attributed to plasmon resonances. These LSPRs appear to be hybridized with the substrate analogously to the proximal and distal plasmon modes of silver nanocubes, indicating that Pd does not prevent coupling and mode splitting^24^,^42^. The proximal LSPR (2) has field intensity penetrating mainly into the higher refractive index Si_3_N_4_ substrate, and is thus lowest in energy, between 1.3 and 2.1 eV (Fig. 2). The distal LSPR (3) has more field intensity away from the substrate, penetrating into the vacuum, than inside the substrate; this mode is consequently higher in energy. Both modes have strong EEL probability at the sharp particle tips, similar to the modes observed for nanocubes using either optical or electron techniques^24^,^42^,^43^,^44^,^45^.

The tilt series shown in Fig. 3 and Supplementary Figs 8–9 support the conclusion that the Pd-rich tips are coupling with the substrate akin to nanocubes and as very recently predicted with finite-difference time-domain (FDTD) calculations for both alloy and pure Au octopods^46^. Coupling with the substrate is obvious from the tilt series: As the particle is tilted, several branches are moved away from being superimposed with the branches touching the substrate, such that they uniquely contribute to the high energy distal resonance (LSPR 3, 1.9 eV). These isolated branches are towards the top (positive tilt) and the bottom (negative tilt) of Fig. 3, and negative tilts, respectively, an increase in tilt corresponding to the top of the substrate moving away from the reader. As expected, the proximal resonance (LSPR 2, 1.55 eV) behaves inversely, with higher intensity where the substrate is closer to the reader (bottom at positive tilts). The two LSPR features, while observed in similar orientations for all particles studied, vary in relative intensity, an observation we have yet to fully elucidate and quantify; particle size, area of contact with the substrate, as well as shape and size of the branches all might play a role. Additional, non-LSPR spectral factors were extracted by NMF. Briefly, spectral factor 1 is the tail of the ZLP, constant far from and decreasing sharply within the particle due to absorption and scattering. Spectral factor 4 is broad with an onset around 2 eV and a relatively flat intensity across the particle; its spectral shape and spatial distribution suggest an interband transition^12^,^13^. Spectral factors 5 and 6 are background noise kept to ensure a full, consistent analysis for all the 11 particles studied.

**Figure 4:**
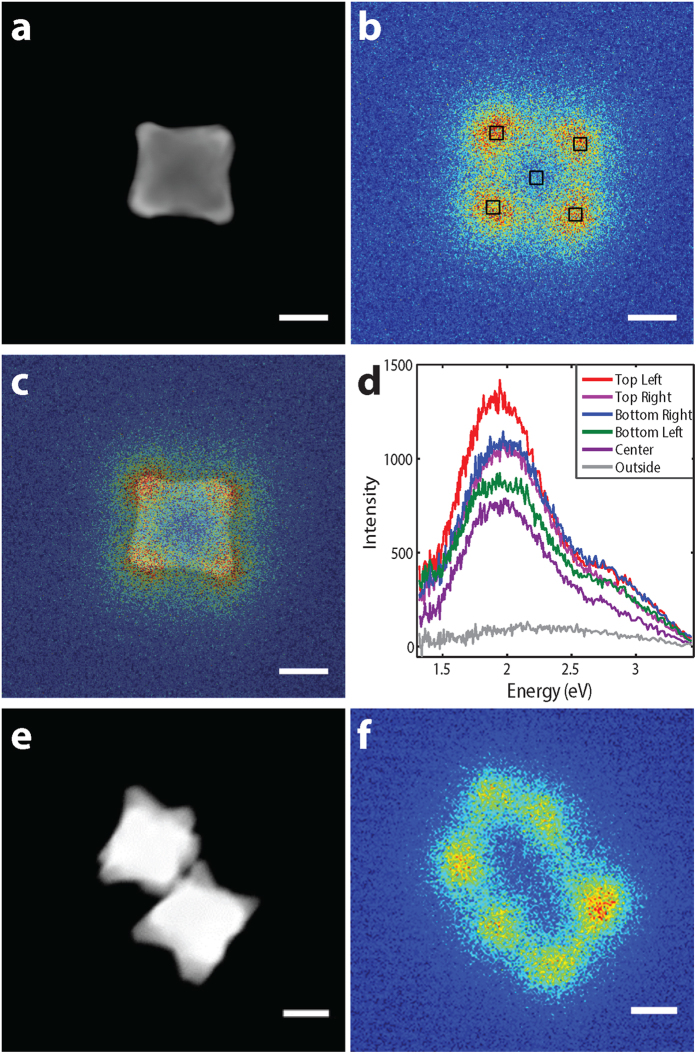
STEM-CL spectroscopy of Au/Pd octopods. (**a**) HAADF-STEM image of a single octopod. (**b**) Panchromatic-CL image of the octopod in **a**. (**c**) Overlay of panchromatic-CL and HAADF-STEM images from **a** and **b**. (**d**) Spectra obtained at the positions marked in **b**. (**e**) HAADF-STEM image of an octopod dimer. (**f**) Panchromatic-CL image of the dimer in **f**. Scale bars, 50 nm.

CL provides complementary information to EELS and is especially useful to identify modes in coupled particles: EELS can excite all LSPRs, while CL only detects bright modes. Panchromatic-CL (all energies acquired, Fig. 4) maps clearly show that Au retains plasmonic properties in the presence of Pd and that modes are localized right at the Pd-rich tips. The CL spectra obtained at various positions on a single 127 nm octopod peak around 2 eV, as expected (Fig. 2, Supplementary Fig. 10); this LSPR is a bright dipolar resonance. The STEM-CL map of an octopod dimer shows the bright bonding LSPR with high CL emission at the tips distant from the interparticle gap, this resonance corresponds to the 1.5 eV LSPR in Fig. 2d. Moreover, little emission is observed from the interparticle gap region, confirming that the LSPR in Fig. 2e is a dark antibonding mode and that Pd-rich nanoparticles can couple akin to their Au counterparts.

Bimetallic nanoparticles with well-defined, sharp, and controllable geometries are promising multifunctional platforms for various applications including optical sensing and catalysis. In this paper, we have shown that single crystalline Au/Pd octopods display plasmonic behavior despite the presence of Pd, a metal not suitable on its own for plasmonic applications. Plasmon modes related to those in nanocubes were observed, and their localized EELS and CL intensity maps suggest localized electric field enhancement at the Pd-rich tips as well as interparticle and substrate coupling, all hallmarks of plasmonic materials. Although the synthetic technique currently does not enable the Au-Pd ratio in the octopod structures to be tuned much beyond 10 atomic % Pd without dramatic changes in morphology^19^, calculated scattering spectra of similarly sized octopods indicate that the LSPRs can be sustained at higher Pd enrichment and with wider Pd tips^46^. Taken together, these results indicate that further studies of bimetallic nanoparticles over a range of compositions are warranted, where a plasmonic core can provide not only sensing, but also well-defined local field enhancement co-localized with catalytically active materials.

## Methods

### Nanoparticle synthesis

Au/Pd octopods were synthesized according to a previously reported method^8^. Au nanoparticle cores were obtained from a seed-mediated reduction of HAuCl_4_ by L-ascorbic acid in the presence of cetyltrimethylammonium bromide (CTAB). Small cores were then coated with a Au/Pd alloy through co-reduction of H_2_PdCl_4_ and HAuCl_4_ by L-ascorbic acid at room temperature. Supplementary Fig. 1 shows a representative sample, and additional bulk composition and extinction information is reported in the Supplementary Discussion and Supplementary Fig. 11. Most of the particles obtained were octopods, with a few larger star-shaped particles derived from twinned right bipyramids. Particles were drop cast from solution on Si_3_N_4_ membrane windows (EELS, EDS, HR-TEM, diffraction, tomography), or carbon-coated grids (CL, tomography).

### Crystallography and diffraction

Electron diffraction and high resolution imaging (Fig. 1, Supplementary Figs 3c,d, 5a–c, Supplementary Movie 2) were performed in a probe-corrected JEOL ARM CFEG operated at 200 kV. CBED patterns taken with the beam perpendicular to one of the underlying cube faces all show 4-fold symmetry attributable to a FCC <100> orientation. Every lattice spacing measured, either via FFT, atomic column distance measurements, or diffraction patterns, fell between the value for Au and that for Pd, confirming alloying. However, the lattice spacing difference between Au and Pd is of the order of the measurement error, such that using it for quantitative compositional analysis is difficult.

### EDS analysis

EDS spectra (Fig. 1, Supplementary Figs 4–5) were acquired with a FEI Titan Chemistem operated at 200 kV, using a Bruker Super-X quad EDS detector. Data analysis was performed in HYPERSPY (available at hyperspy.org). The relative X-ray intensity was obtained by first subtracting the background, then integrating the peak intensity over an appropriate energy range, and finally dividing the integrated peak intensity by the total X-ray count. This process is used to separate the effects of thickness and composition, as shown in Supplementary Fig. 4. To produce the images in Fig. 1 and Supplementary Fig. 5, areas outside the particle were set to zero relative intensity by applying an intensity threshold for the X-ray peak studied in order to avoid large fluctuations due to the division of two small noise-dominated numbers.

### STEM-EELS and CL

STEM-EELS (Figs 2a-3, Supplementary Figs 7–9) was performed on a probe corrected, monochromated FEI Titan Themis operated at 200 kV, equipped with a X-FEG electron gun and a Wien filter monochromator, or (Fig. 2d,e) a JEOL ARM CFEG operated at 200 kV. EELS spectra were acquired with a Gatan GIF Quantum ERS energy-loss spectrometer (Figs 2a,3, Supplementary Figs 7–9) or a Gatan Enfinium ER energy-loss spectrometer (Fig. 2d,e). SIs measuring 164 × 164 pixels were acquired at +30°, 0°, and −30° for the particle presented in Figs 2a-3 and Supplementary Figs 7–8. A SI measuring 128 × 207 pixels was acquired at 0° for the dimer in Fig. 2d,e.

Energy-filtered TEM (EFTEM, or in this case “EF-STEM”) images were obtained by integrating the STEM-EELS intensity over a 0.2 eV window centered at 1.5 and 2.3 eV in Fig. 2 and 1.65 and 2.5 eV in Supplementary Fig. 7. The EFTEM energy window values were selected by determining the apparent peak position in the EEL spectra. Simulated EFTEM images at high energy (>5 eV) closely resemble the spatial distribution of the interband transitions, however it is difficult to assess the interband nature as the spectral shape is not accessible with simulated EFTEM.

The multidimensional data array (164 × 164 pixels region of interest, 2048 energy channels, 3 tilts for the particle in Figs 2a-3, Supplementary Fig. 7) was analyzed using blind source separation of decomposed modes from non-negative matrix factorization performed in HYPERSPY^24^,^39^,^47^. This approach decomposes the intrinsically redundant information of a spectrum image (SI) into a number of spectral components (spectral factors) that are multiplied by different coefficients (loadings) at each pixel to best fit the SI. The factors were not assigned specific peak shapes. The spectra analyzed were cropped from 0.3 to 5 eV and all three tilts were processed simultaneously. The two distinct particles in Fig. 3 and Supplementary Fig. 9 were present on the same Si_3_N_4_ grid and investigated in the course of a single experiment, making the tilting behavior (axis orientation and tilt direction) directly comparable. Results reported in Fig. 4 and Supplementary Fig. 10 were obtained using a cathodoluminescence holder (Vulcan holder, GATAN, Inc) inserted into a JEOL 2100F STEM operated at 200 kV.

## Additional Information

**How to cite this article**: Ringe, E. *et al.* Resonances of nanoparticles with poor plasmonic metal tips. *Sci. Rep.* **5**, 17431; doi: 10.1038/srep17431 (2015).

## Supplementary Material

Supplementary Movie 1

Supplementary Movie 2

Supplementary Information
