# Supplementary material for: Resonances of nanoparticles with poor plasmonic metal tips
Source: Sci Rep. 2015 Nov 30;5:17431. doi: 10.1038/srep17431 (PMC4663493; doi:10.1038/srep17431)
Supplement: Supplementary Information [file srep17431-s3.pdf]

Supplementary Information

Resonances of nanoparticles with poor plasmonic metal tips

Emilie Ringe,[1] Christopher J. DeSantis,[2] Sean M. Collins,[3] Martial Duchamp,[4] Rafal E. Dunin-Borkowski,[4] Sara E. Skrabalak,[2] Paul A. Midgley[3]*

1. Department of Materials Science and NanoEngineering, Rice University, 6100 Main St., Houston TX 77005, USA

2. Department of Chemistry, Indiana University, 800 E. Kirkwood Ave., Bloomington, IN 47405, USA

3. Department of Materials Science and Metallurgy, University of Cambridge, 27 Charles Babbage Road, Cambridge CB3 0FS, UK

4. Ernst Ruska-Centre for Microscopy and Spectroscopy with Electrons (ER-C) and Peter Grünberg Institut 5 (PGI-5), Forschungszentrum Jülich GmbH, D-52425 Jülich, Germany

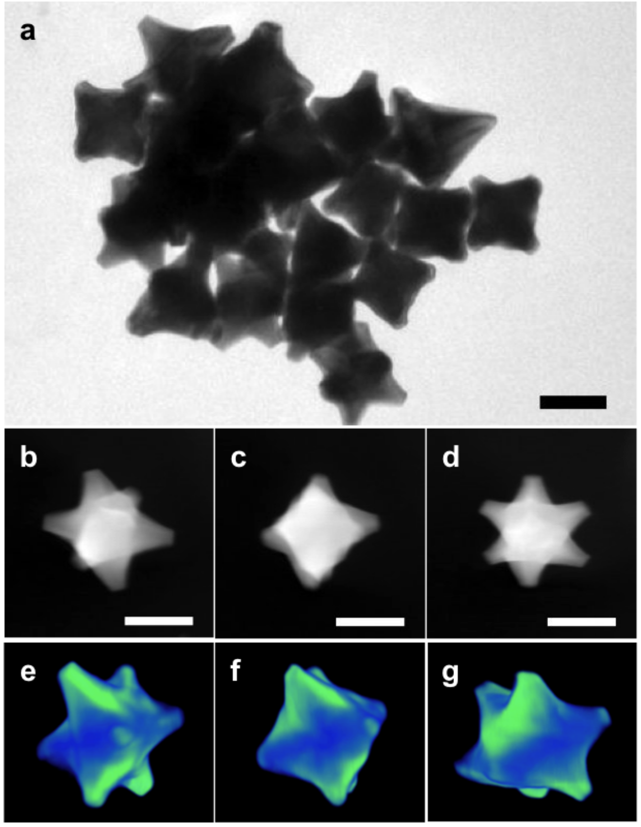

Supplementary Figure 1. Structure of Au/Pd octopods. (a) Bright field TEM of the sample studied; the average tip-to-tip (face diagonal) distance is 118 (±8) nm with a tip thickness of 21 (±3) nm. (b-d) HAADF-STEM images of an octopod at -50°, 0°, and +50°. (e-g) Snapshots of the 3-dimensional reconstruction obtained from electron tomography of the particle shown in b-d (from Supplementary Movie 1). Scale bars, 100 nm.

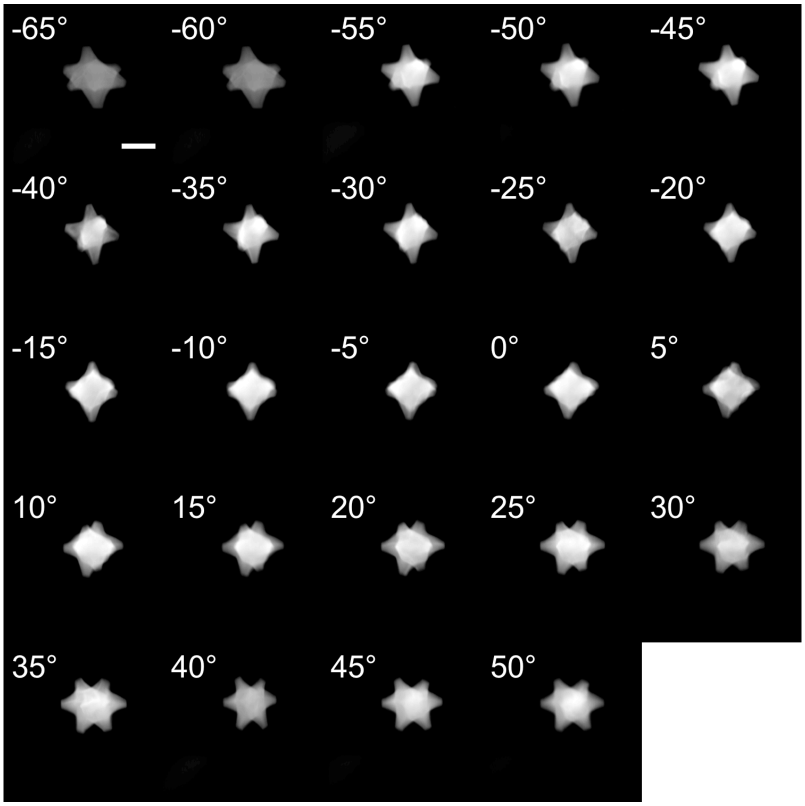

Supplementary Figure 2. HAADF-STEM tilt series for the octopod shown in Supplementary Movie 1 and Supplementary Fig. S1. Scale bar, 100nm; the scale is the same for all the pictures.

Supplementary Movie 1 (available as a separate download) Reconstructed volume for the particle shown in Supplementary Fig. S1-S2. The colors are solely for contrast.

Supplementary Movie 2 (available as a separate download) Convergent beam electron diffraction mapping of the particle in Fig. 1a.

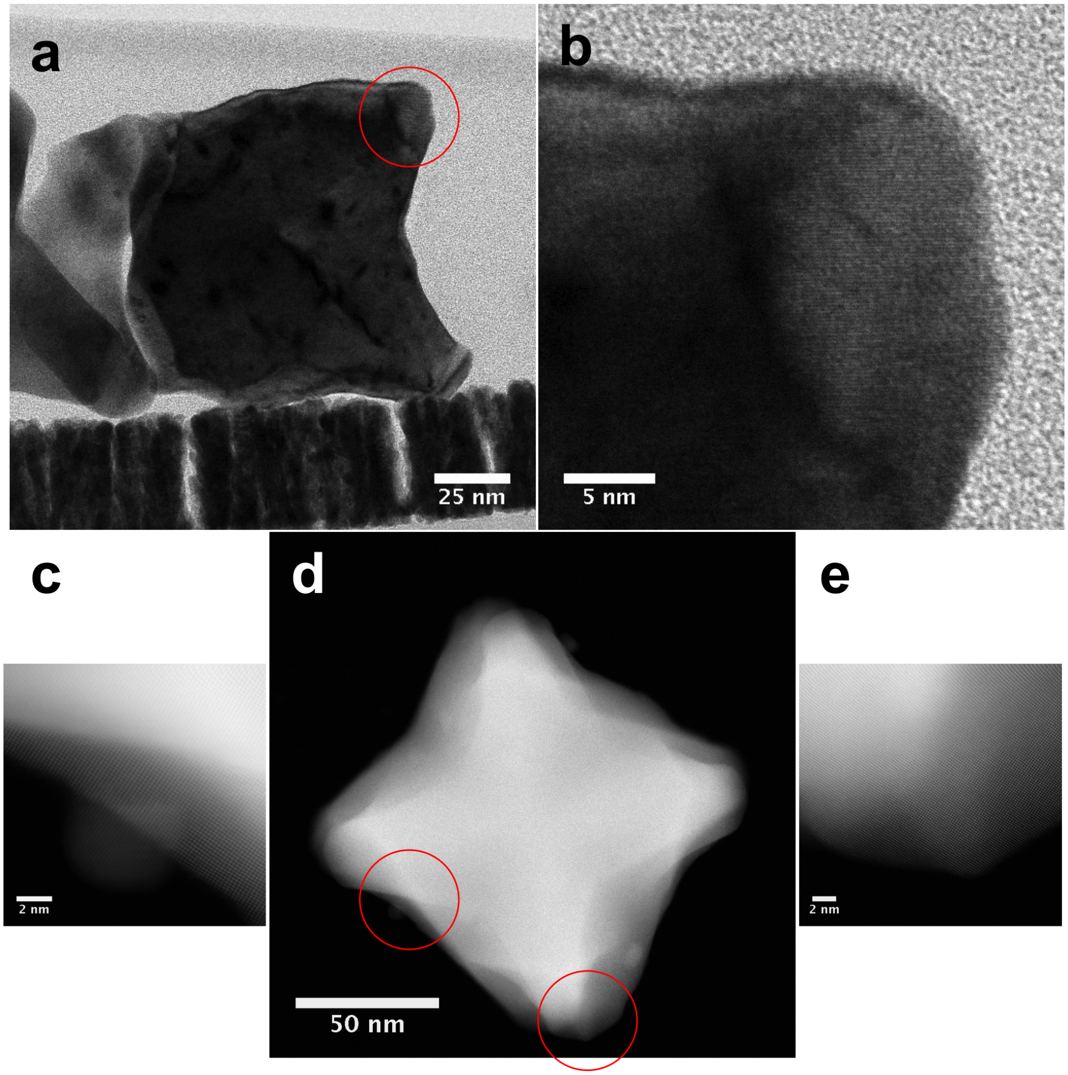

Supplementary Figure 3. Additional atomic resolution images of octopods highlighting the absence of defects and the single crystalline nature of the branches. (a) Bright field STEM image of an octopod cut in half by an focused ion beam, adapted with permission from ref.[1] (b) Higher magnification of the region shown in a, the horizontal lattice fringes correspond to {200} planes with a distance of ~0.2 nm. (d) HAADF-STEM images of an octopod. (c, e) Higher magnification of the regions marked in d.

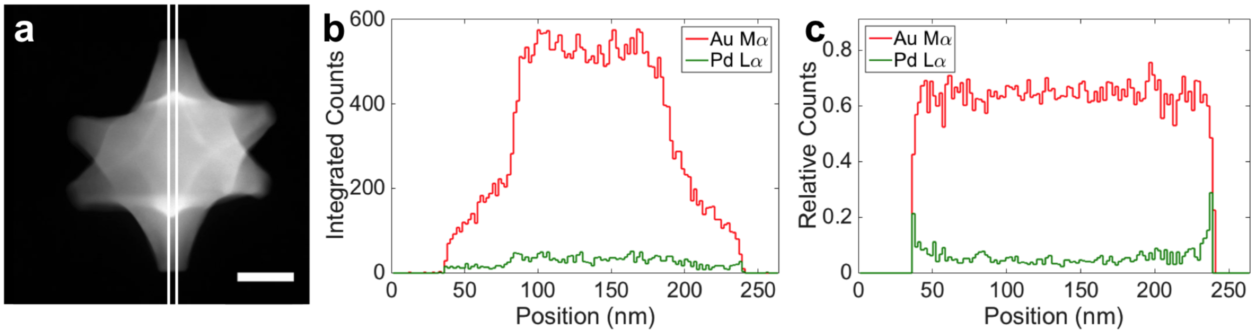

Supplementary Figure 4. Additional EDS linescan data for a single octopod tilted -30°. (a) HAADF-STEM image. (b) Background subtracted, integrated counts for Au M_α and Pd L_α lines. The two broad peaks (~100 and ~180 nm) correspond to the position of two branches, one above and one below the particle core. (c) Background subtracted, integrated and normalized counts for Au M_α and Pd L_α lines. Scale bars, 50 nm.

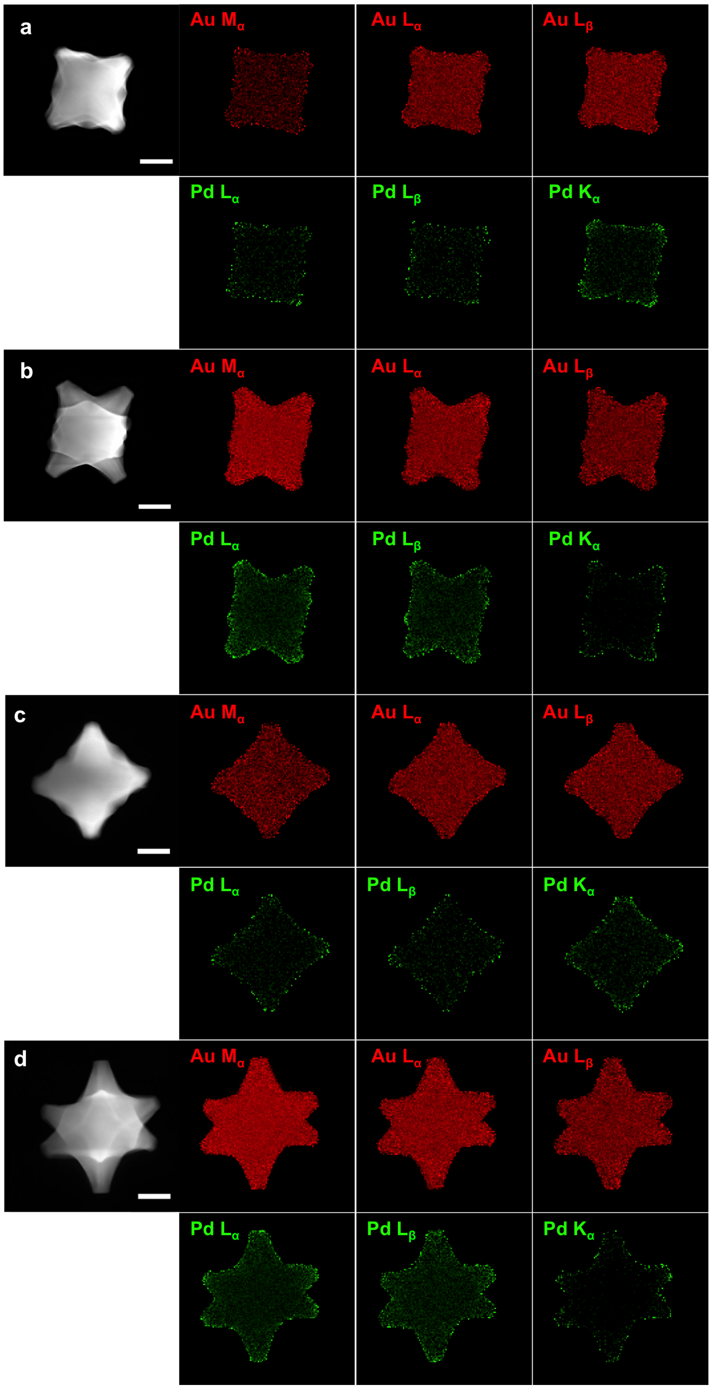

Supplementary Figure 5. HAADF-STEM and EDS maps of two octopods. (a-b) Octopod in Supplementary Fig. 8, tilted 0° and +30°. (c-d) Octopod in Figs. 2a, 3, and Supplementary Fig. 6-7, tilted 0° and -30°. Scale bars, 50 nm. The EDS and STEM images have the same scale.

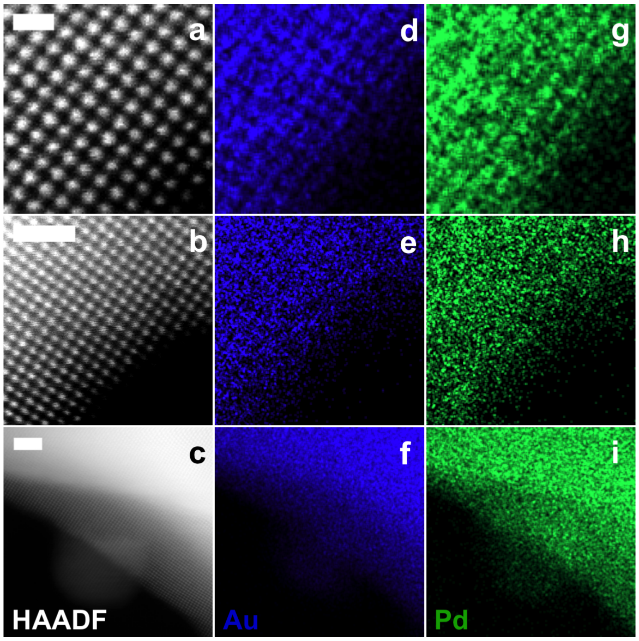

Supplementary Figure 6. High resolution EDS analysis of Au/Pd octopods. (a-c) HAADF-STEM images. (d-e) Au intensity from the M_{α} and L_{α} line. (g-h) Pd intensity from the L_{α} and L_{β} lines. Scale bars, 0.5 nm for a, 1 nm for b, 2 nm for c. The EDS and STEM images have the same scale.

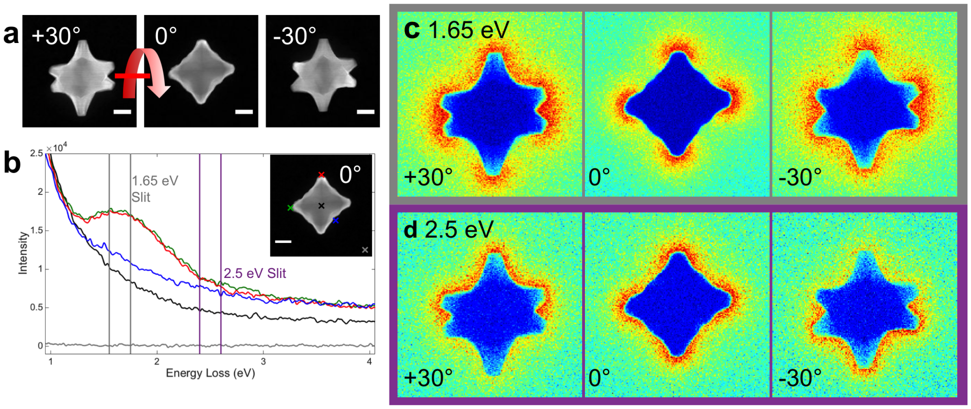

Supplementary Figure 7. Simulated EFTEM images and EELS spectra. (a) HAADF-STEM images at various tilts, the horizontal red line indicates the tilt axis. (b) EELs spectra integrated over 11X11 pixels at the locations indicated in the inset, as in Fig.4. (c-e) Simulated EFTEM images obtained from integrating the intensity using a virtual 0.2 eV slit centered at the apparent peak position, 1.65 eV, as well as the tail of the plasmon, 2.5 eV. The 2.5 eV data is consistent with the conclusion that the plasmon is hybridized with the substrate in a proximal and distal configuration. Scale bar, 50 nm. The EELS and STEM images have the same scale.

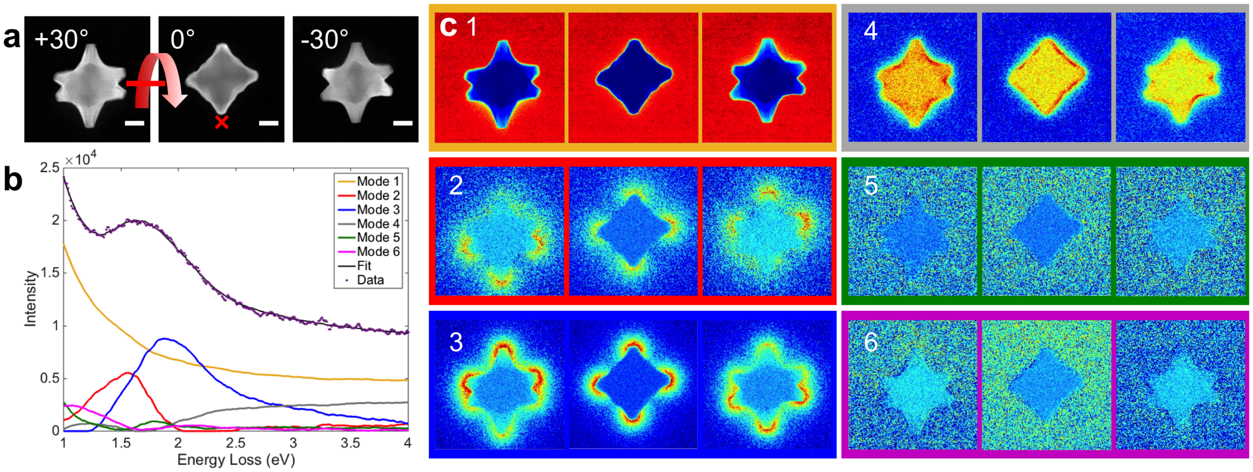

Supplementary Figure 8. Full statistical analysis of EELS results on a single Au/Pd octopod, the same particle shown in Fig. 3. (a) Dark field STEM images obtained concurrently to the EELS SI. (b) Spectral factors extracted from NMF as well as the fit of the raw data using the EELS response at the position marked in a. (c) Loadings for each of the spectral factors (1-6), representing the intensity of each factor in b at every pixel of the image. Spectral factor 1 is the tail of the ZLP, 2 is the low-energy proximal (into the substrate) LSPR, 3 is the high-energy distal (away from the substrate) LSPR, 4 is due to interband transitions, 5 and 6 are noise included to insure a full and complete analysis. Scale bars, 50 nm. The EELS and STEM images have the same scale for each tilt.

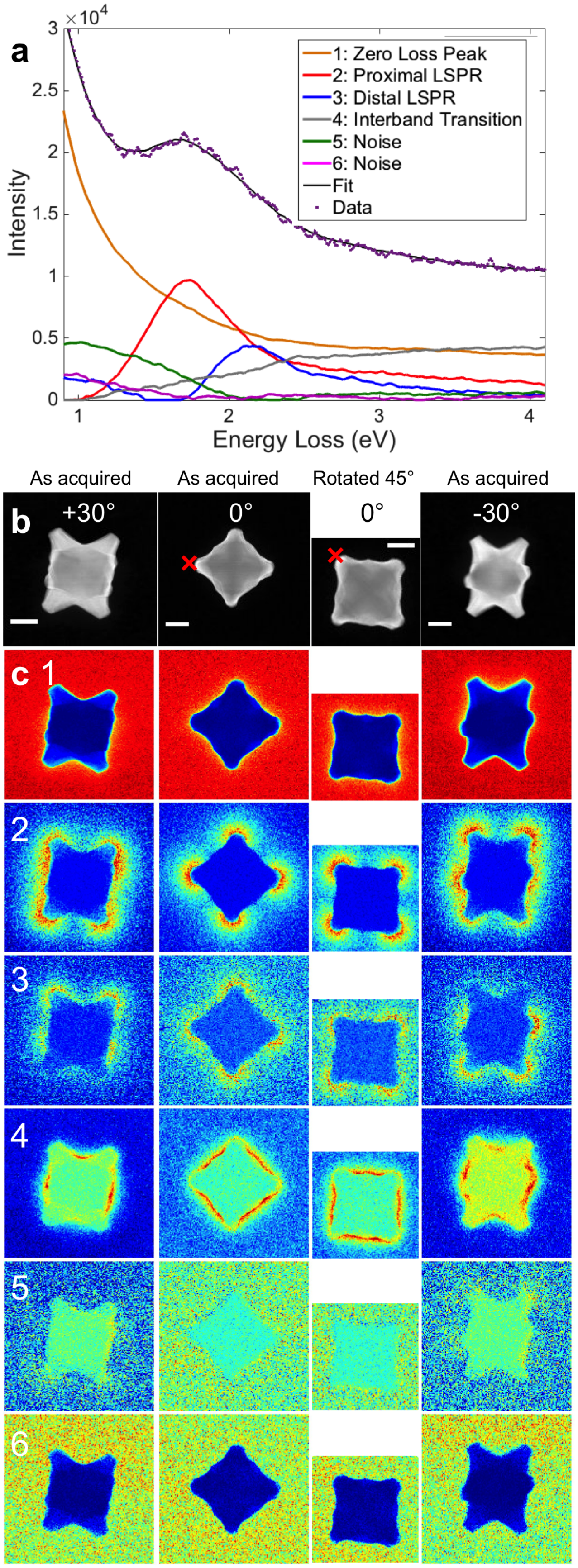

a
$\times10^4$
Intensity
Energy Loss (eV)
1: Zero Loss Peak
2: Proximal LSPR
3: Distal LSPR
4: Interband Transition
5: Noise
6: Noise
Fit
Data
As acquired
As acquired
Rotated 45°
As acquired
b
+30°
0°
0°
-30°
c
1
2
3
4
5
6

Supplementary Figure 9. Full results of NMF for an additional single octopod rotated approximately 45° from the octopod in Figs. 2a-3 and Supplementary Figs. 7-8. (a) Spectral factors extracted from NMF as well as the fit of the raw data using the EELS response at the position marked in b. (b) Dark field STEM images obtained concurrently to the EELS SI. (c) Loadings for each of the spectral factors (1-6), representing the intensity of each factor in b at every pixel of the image. Spectral factor 1 is the tail of the ZLP, 2 is the low-energy proximal (into the substrate) LSPR, 3 is the high-energy distal (away from the substrate) LSPR, 4 is due to interband transitions, 5 and 6 are noise and remaining ZLP contributions included to insure a full and complete analysis. The field intensity distribution orientation for the LSPR modes matches well that of Fig.3. The first, second, and fourth columns in both b and c are "as acquired", while the third column shows data rotated 45° to match the orientation of the first and fourth columns. Scale bars, 50 nm; the EELS and STEM images have the same scale.

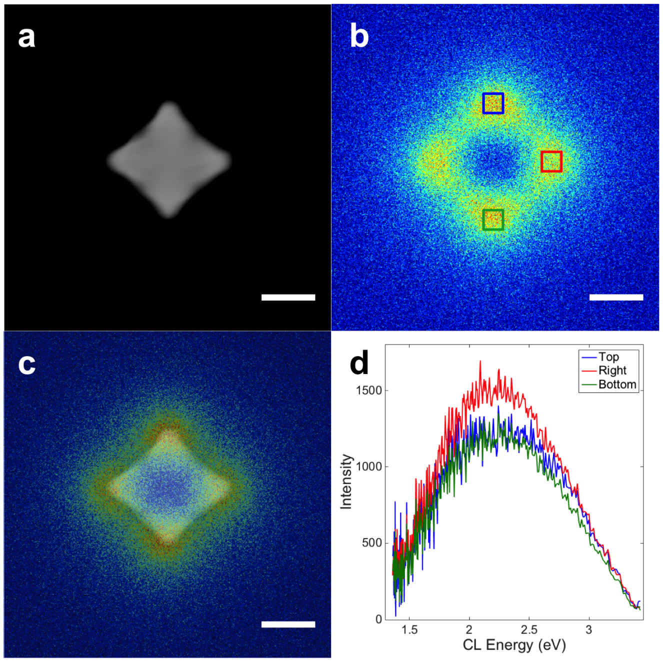

Supplementary Figure 10. Additional STEM-CL data from of a single Au/Pd octopod measuring 100 nm tip to tip. (a) HAADF-STEM image, (b) Panchromatic-CL image. (c) Overlay of panchromatic-CL and HAADF-STEM images. (d) Spectra obtained at the positions marked in b. Scale bars, 50 nm.

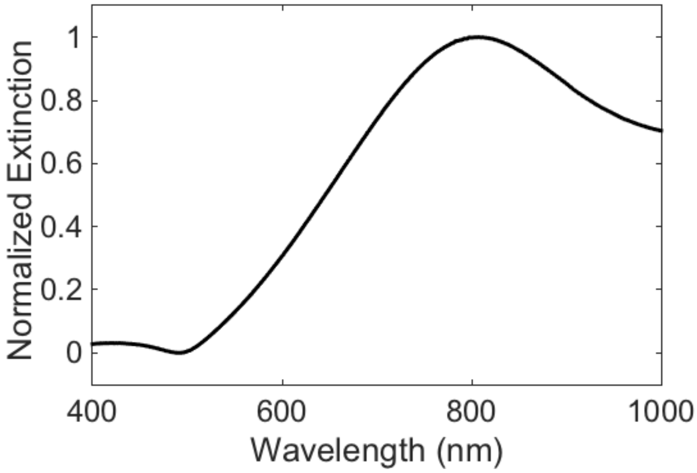

Supplementary Figure 11. UV-Vis spectra of octopod solution in ethanol.

Supplementary Methods

Tomographic reconstructions (Supplementary Fig. 1 & Supplementary Movie 1) were obtained from HAADF-STEM tilt series acquired (Supplementary Fig. 2) on a FEI Tecnai FEG operated at 200 kV using a simultaneous iterative reconstruction technique (SIRT) in the software Inspect 3D (FEI Company). The voxel projection visualization of reconstructed volume was obtained in Avizo Fire (Visualization Science Group), without any cropping of the area surrounding the particle.

Supplementary Fig. 6 shows the net integrated signal of the Au M_{α} and L_{α} and Pd L_{α} and L_{β} data acquired on a JEOL ARM Cold FEG operated at 200 kV, as an additional and higher resolution confirmation of the presence of Pd at the sides and tips of the particle. The octopod analyzed in Supplementary Fig. S6 is the same as that in Supplementary Fig. 3c-e.

The "As-aquired" SI and HAADF-STEM data at 0° in Supplementary Fig. 9 is rotated 45° with respect to the tilted SI (+30° and -30° tilt). The SI and HAADF (164X166 pixels) were collected at 0° prior to a 45° rotation of the sample. For ease of readability, the HAADF-STEM and loading maps in the third column of Supplementary Fig. 9 were reproduced from the second column with the appropriate 45° image rotation and crop to match the orientation of the +30° (142X142 pixels) and -30° (164X164 pixels) SI subsequently acquired. The SI images were cropped to 142X142 pixels, and the spectra were cropped from 0.3 to 5 eV, all three tilts were processed simultaneously and the factors were not assigned fixed peak shapes.

Supplementary Discussion

The blue shift observed in vacuum/Si_3N_4 membrane (EELS) compared to water (bulk extinction measurements[2]) is equivalent to that caused by a change in refractive index (RI) of 0.16 RI units, as calculated from the RI sensitivities and LSPR energies obtained optically.[2] A RI of ~1.17 (1.333 for water minus 0.16) is well justifiable: vacuum and Si_3N_4 have RIs of 1, and ~2.05, respectively, and the particle interacts mostly with the former, given only the tips of four branches are touching the substrate. Other effects could but appear not to play a role, namely the size- and shape-dependence of the RI sensitivity, the error associated with determining the LSPR energy in bulk measurements, and the expected small redshift of the near-field peak with respect to the far-field maximum.[3]

For this sample, a bulk composition of 7.9% Pd atoms was determined by inductively coupled plasma-optical emission spectroscopy (ICP-OES, Perkin-Elmer Optima 8300).

A previously derived equation[4] was used to estimate the volume of a single octopod ($7.63*10^5$ nm^3), a value used in the calculation of the molar absorption coefficient, $9.85*10^{11}$ M^{-1} cm^1.

References

1 Ringe, E. Nanocrystalline materials: recent advances in crystallographic characterization techniques. *IUCrJ* **1**, 530-539, doi:10.1107/S2052252514020818 (2014).

2 DeSantis, C. J. & Skrabalak, S. E. Size-controlled synthesis of Au/Pd octopods with high refractive index sensitivity. *Langmuir* **28**, 9055-9062, doi:10.1021/la3002509 (2012).

3 Zuloaga, J. & Nordlander, P. On the energy shift between near-field and far-field peak intensities in localized plasmon systems. *Nano Lett*. **11**, 1280-1283, doi:10.1021/nl1043242 (2011).

4 DeSantis, C. J. *et al*. Shaping the synthesis and assembly of symmetrically stellated Au/Pd nanocrystals with aromatic additives. *Nano Lett*. **14**, 4145-4150, doi:10.1021/nl501802u (2014).
